# High‐temperature technology survey and comparison among incineration, pyrolysis, and gasification systems for water resource recovery facilities

**DOI:** 10.1002/wer.10715

**Published:** 2022-04-07

**Authors:** Lloyd J. Winchell, John J. Ross, Dominic A. Brose, Thaís B. Pluth, Xavier Fonoll, John W. Norton, Katherine Y. Bell

**Affiliations:** ^1^ Brown and Caldwell St. Paul Minnesota USA; ^2^ Brown and Caldwell Troy Michigan USA; ^3^ Metropolitan Water Reclamation District of Greater Chicago Cicero Illinois USA; ^4^ Great Lakes Water Authority Detroit Michigan USA; ^5^ Brown and Caldwell Nashville Tennessee USA

**Keywords:** air emissions, energy, gasification, incineration, permitting, PFAS, pyrolysis, residuals, wastewater

## Abstract

Solids from wastewater treatment undergo processing to reduce mass, minimize pathogens, and condition the products for specific end uses. However, costs and contaminant concerns (e.g., per‐ and polyfluoroalkyl substances [PFAS]) challenge traditional landfill and land application practices. Incineration can overcome these issues but has become complicated due to evolving emissions regulations, and it suffers from poor public perception. These circumstances are driving the re‐emergence of pyrolysis and gasification technologies. A survey of suppliers was conducted to document differences with technologies. Both offer advantages over incineration with tailored production of a carbon‐rich solid, currently less stringent air emission requirements, and lower flue gas flows requiring treatment. However, incineration more simply combines drying and thermal processing into one reactor. Equipment costs provided favor pyrolysis and gasification at lower capacities but converge with incineration at higher capacities. Long‐term operational experience will confirm technology competitiveness and elucidate whether pyrolysis and gasification warrant widespread adoption.

**Practitioner Points:**

- Pyrolysis and gasification systems are gaining traction in the wastewater industry with several full‐scale installations operating, in construction, or design
- Several advantages, but some disadvantages, are considered in comparison with incineration
- Organic contaminants, including PFAS, will undergo transformation and potentially complete mineralization through each process

## Graphical abstract

Solids generated during wastewater treatment require dedicated processing, dictated in part by intended end use. Landfill costs, land application challenges, and unregulated contaminant concerns have driven wastewater treatment providers to consider alternative solids handling processes that reduce the final residual mass and destroy polluting chemicals. Pyrolysis and gasification technologies are emerging in the market, providing a potential solution to the current end use challenges, and offer certain advantages over the historically proven incineration technology.

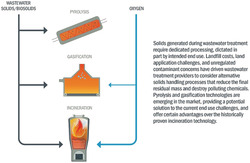

## INTRODUCTION

Solids generated or captured during wastewater treatment typically undergo processing before beneficial use or ultimate disposal. These processes aim to reduce the mass and volume of the solids, and therefore costs and logistics for the end use or change in custody. Historically, the wastewater industry referred to these materials as sewage sludge, but contemporary nomenclature prefers the term biosolids if processing meets federal and state requirements for beneficial reuse (WEF, 2021).

**FIGURE 1:**
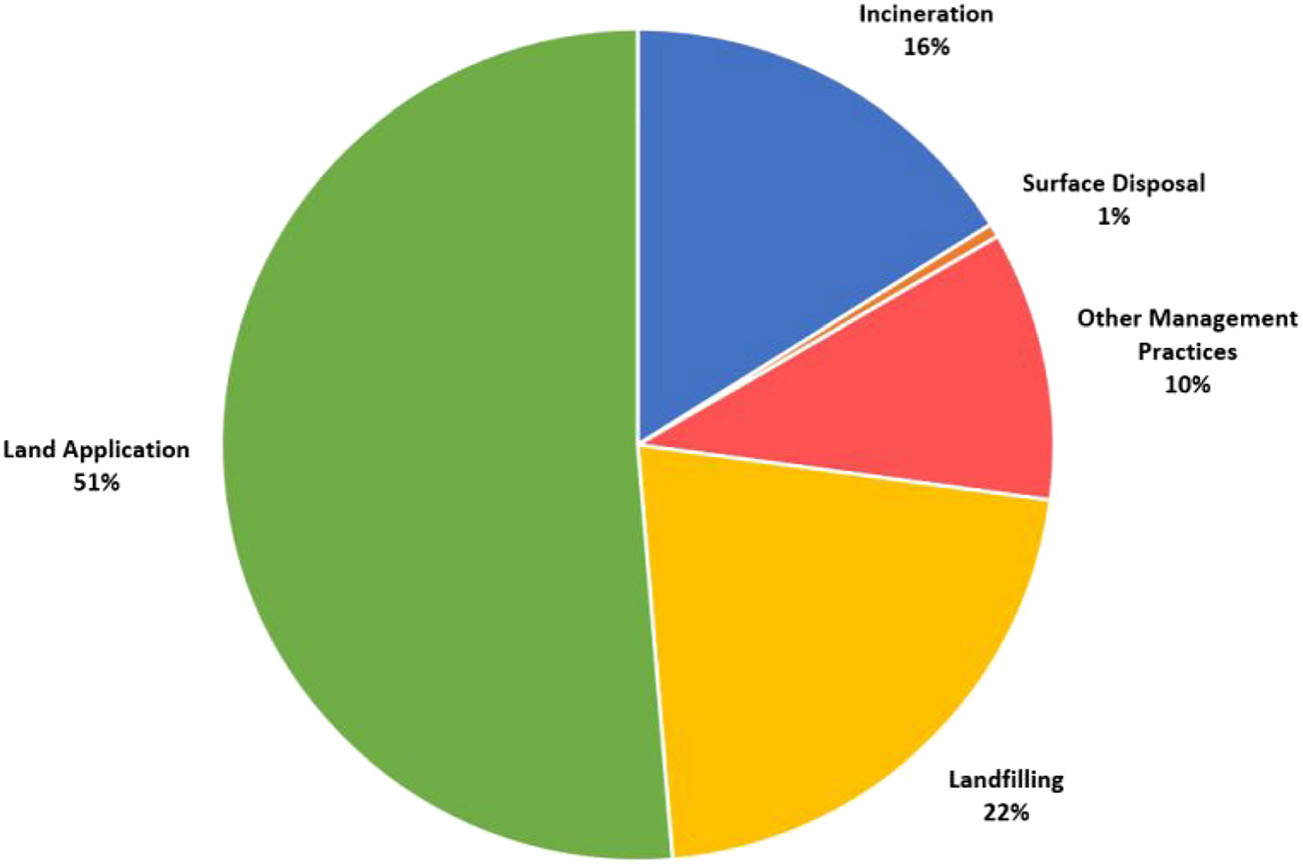
USEPA reported biosolids end use and disposition proportions in 2019 (USEPA, 2021a)

As shown in Figure 1, the United States Environmental Protection Agency (USEPA) estimated that 4.75 million dry tonnes (5.2 million dry tons) of “biosolids” were produced in the United States during 2019 by water resource recovery facilities (WRRFs) treating 3.8 megaliters per day (one million gallons per day) or more (USEPA, 2021a). WRRF operators land applied over half of this material and landfilled almost one fourth. However, these heavily relied upon outlets for sludge or biosolids face increasing challenges from shifts in acceptable land‐use practices, regulations, and unregulated chemicals.

As population increases in the United States (Vespa et al., 2018), urban sprawl (USDA, 2020) impacts land uses. These trends result in loss of potential sludge or biosolids land application sites when coupled with a general public aversion to reusing human waste products (Collins, 2019; Slaughter, 2013). Landfilling options also continue to decline due to closures and new facility permitting challenges (USEPA, 1994). More recently, landfill tipping fees in the United States have increased (EREF, 2021), most prominently in densely populated areas where sludge and biosolids production is comparatively high. Considered alone, these drivers create a groundswell for alternative sludge and biosolids end use, and the ever‐increasing interest in per‐ and polyfluoroalkyl substances (PFAS) and other emerging pollutants compound these challenges (Boxall et al., 2012; Kinney et al., 2006; Navarro et al., 2018; Sepulvado et al., 2011; Walters et al., 2010; Winchell, Wells, et al., 2022).

Incineration of sludge and biosolids is a widely applied and accepted technology for addressing this suite of challenges because it provides a high level of control for municipalities. However, obtaining and maintaining permits to construct and operate facilities comes at a significant cost and resource commitments in addition to relatively complex operation and maintenance requirements (WEF, 2009). These complications create an opportunity for alternative technologies to enter the industry.

Pyrolysis and gasification systems offer incineration alternatives while achieving similar objectives for mass and handling cost reductions. In 2019, roughly 16% of the sludge generated in the United States was processed through an incinerator (Figure 1). In the authors' experience, most operating sewage sludge incinerators (SSIs) have close to, or more than, 20 years of operation—the typical service life assumed for mechanical equipment. In response, several WRRF owners already plan to evaluate next‐generation thermal treatment technologies in the context of the compounding drivers previously noted.

An objective comparison among incineration, pyrolysis, and gasification provides valuable knowledge for the wastewater industry. For almost 100 years, incineration has been used at WRRFs to process sludge or biosolids into sterilized ash, and many facilities also recover heat to facilitate the process or export energy (WEF, 2009). Recent changes in the Sewage Sludge Incineration emissions regulations, 40 C.F.R. § 60 (USEPA, 2011) have, in part, led to the closure of several SSIs. Permitting obstacles and the process complexity of incineration have also prompted the industry to seek alternatives. A comprehensive comparison includes assessment of capital costs, energy requirements, permitting conditions, system complexity, air emissions, and residual product characterization.

**FIGURE 2:**
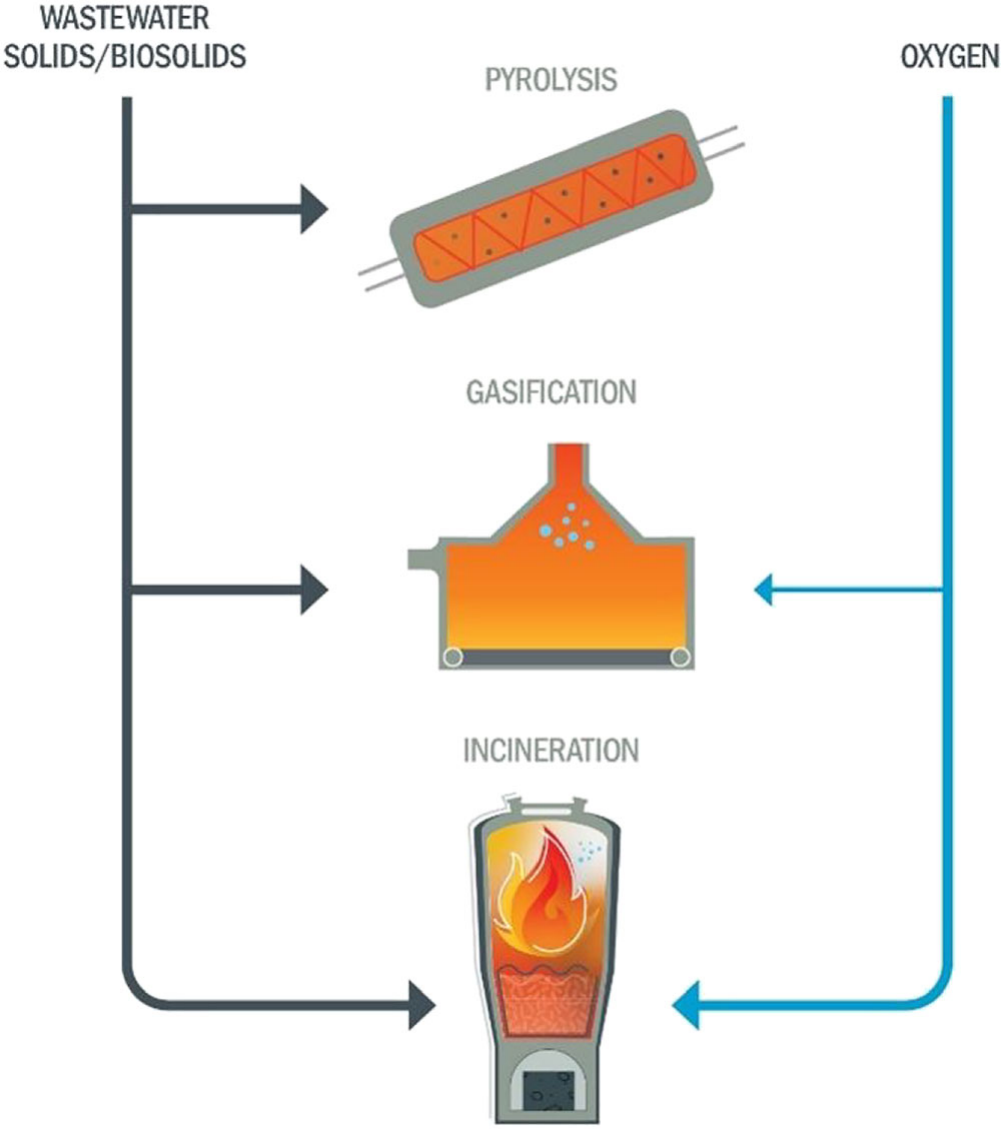
Commercially viable high‐temperature technologies available for sludge and biosolids treatment illustrating the basic difference in application of oxygen

This paper provides a detailed description of pyrolysis and gasification technologies in the context of incineration as a base case practice (Figure 2), focusing on application in the United States. Comparing the thermochemistry, processing train general arrangements, state and disposition of residuals following treatment, unregulated chemical fate, permitting, and equipment capital costs are described. As a generalized resource, this discussion is provided for potential end users to facilitate their site‐specific evaluation of alternative high‐temperature technologies for sludge or biosolids treatment.

## TECHNOLOGY COMPARISON

Incineration offers a proven high‐temperature process that significantly reduces solid residuals transported off‐site from WRRFs. The promise exists for emerging pyrolysis and gasification systems to achieve such benefits; however, historical challenges around pyrolysis and gasification have left the high‐temperature processing market primarily to incineration at WRRFs. With the current pressures on land application and landfilling of sludge and biosolids, pyrolysis and gasification are receiving renewed interest as alternative high‐temperature processes. Thus, they deserve an objective comparison with incineration.

Pyrolysis and gasification can convert complex organic feedstocks into gaseous or liquid fuels that are more readily stored or used in combustion applications. Further, the carbon‐rich product, called char or biochar, appeals to WRRFs given their properties for agricultural and industrial applications. While most applications using these technologies exist in the private industry and agriculture sectors, several new installations have been implemented, and several others will soon be operating at WRRFs. Currently, two installations are documented to be in operation: one pyrolysis and one gasification. After a long‐term demonstration project, a third application (gasification) has recently shut down. In addition, five technology suppliers known to the authors are in the process of designing or constructing several facilities across North America. See Winchell, Ross, et al. (2022) for a more in depth discussion on these topics.

Incineration was limited to a fluidized bed furnace (FBF) system for this comparison. Multiple hearth furnaces (MHF) still command a significant portion of the currently operating SSIs. However, their supplementary fuel requirements and comparably higher emissions have not allowed them to compete with FBFs in recent decades (Winchell et al., 2021). An emerging approach that couples a dryer with a combustion furnace is being marketed for PFAS destruction, but there are limited installations globally, one in the United States and five in Europe, and have not been included in this comparison because an overview has been provided elsewhere (Viswanathan et al., 2020). Finally, other furnaces have previously been applied and may still co‐fire sludge or biosolids but were not considered commercially relevant for this comparison. For additional details on these types of furnaces, an in‐depth description is provided in Winchell et al. (2021).

One FBF incinerator, three pyrolysis, and two gasification system suppliers provided information for this comparison. Therefore, detailed information is attributed to correspondence with these system suppliers unless otherwise stated.

### Differentiating reactions

The primary differentiator, outside of temperature and products, between pyrolysis, gasification, and incineration processes is the amount of oxygen introduced into the high‐temperature reactor (Figure 2). Pyrolysis thermochemically converts organic feedstock to carbon‐rich char and a hydrocarbon‐rich off‐gas in the absence of oxygen, typically at temperatures between 300°C and 750°C which may overlap torrefaction on the lower end (Basu, 2013; Bridle & Pritchard, 2004). Gasification processes further refine the char and off‐gas from a pyrolysis step using a gasifying medium (such as air, oxygen [O_2_], or steam [H_2_O]) and often reach temperatures of 800°C–1000°C (Ahmad et al., 2016; Basu, 2013). In the presence of adequate oxygen, the primary constituents of sludge and biosolids—carbon (C), hydrogen (H), oxygen (O), nitrogen (N), and sulfur (S)—are combusted to thermodynamically stable end products. Reaction 1 summarizes this unbalanced reaction for complete theoretical combustion, using typical values of the combustible fraction from untreated mixed primary and waste‐activated sludges (Albertson, 1992).

(1) $$C_{0.57}H_{0.07}O_{0.30}N_{0.05}S_{0.01}+O_{2}\to {\mathrm{CO}}_{2}+H_{2}O+N_{2}+{\mathrm{SO}}_{2}$$

Because incinerators utilize combustion, the reactions are predominantly exothermic and result in a net heat release. The heat value can be measured directly using bomb calorimeter tests. In the absence of specific test data, several empirical equations may be used to assess heat values, generally ranging from 23,000 to 26,000 kJ/kg of dried combustible solids (Albertson, 1992; Niessen, 2002; WEF, 2009). Thus, despite the heavy water evaporation burden, sludge or biosolids FBF incineration systems can often operate without supplementary fuel to maintain combustion reactions and achieve temperatures needed for emissions control.

### General arrangement

**FIGURE 3:**
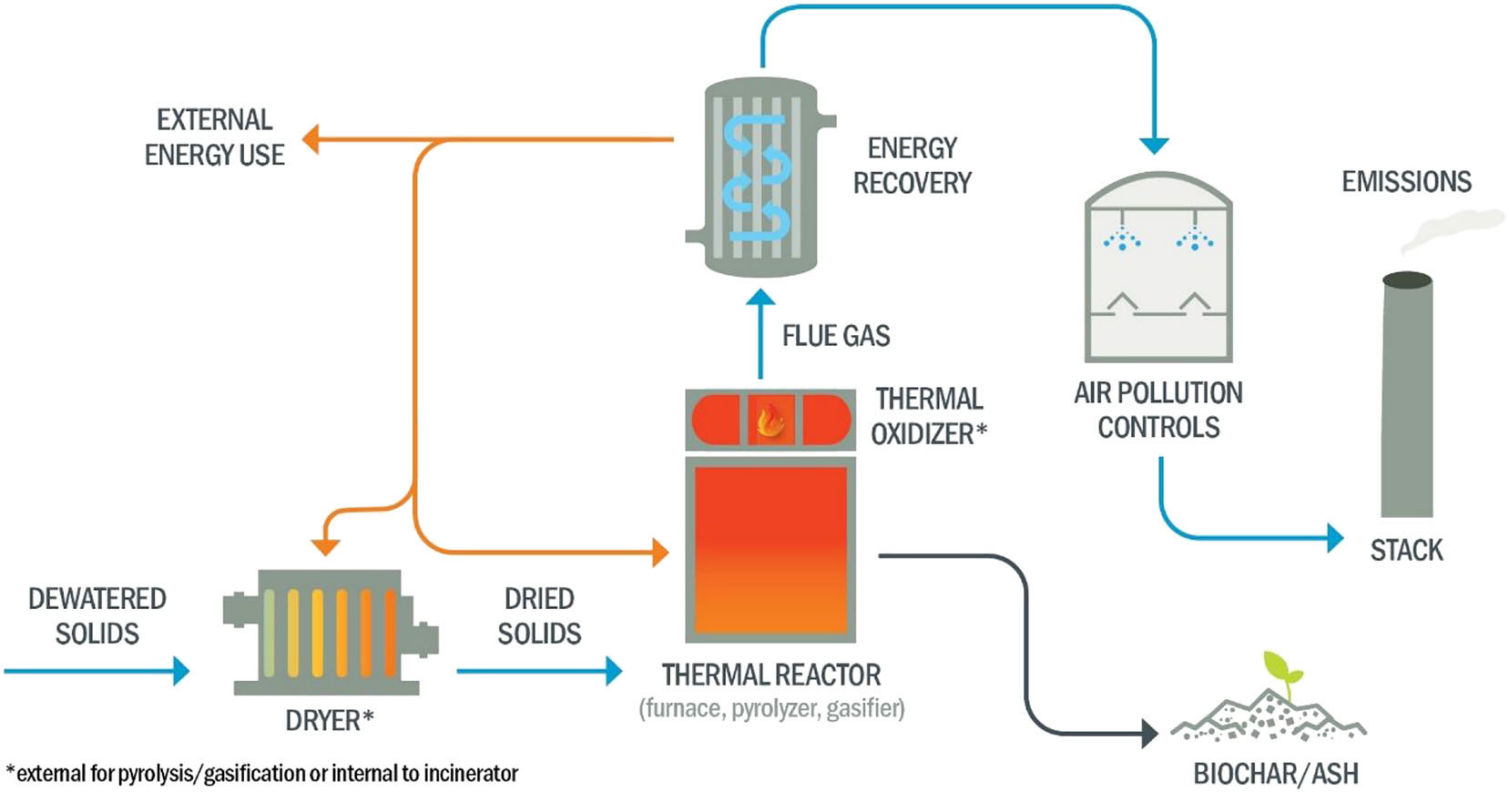
Basic thermal processing schematic for high‐temperature processes for sludge or biosolids applications indicate the same generalized approach for all alternatives

The general arrangement of the process train for pyrolysis, gasification, and incineration is similar. Figure 3 depicts the sub‐processes for thermally treating sludge or biosolids and indicates the processing train is categorically similar among the three technologies, with a few exceptions. The common input to all three technologies is dewatered sludge or biosolids. Typical outputs include gas‐phase emissions from the stack and solid residuals (i.e., char or ash). Table 1 summarizes the fundamental differences among these three processes.

**TABLE 1:** Fundamental differences between high‐temperature thermal processes

	Incinerator (FBF)	Gasification	Pyrolysis
Dryer required	No	Yes	Yes
Air/oxygen requirement in main thermal reactor	Greater than stoichiometric amount	Less than stoichiometric amount	None
Separate thermal oxidizer	No	Yes	Yes
Off‐gas reuse potential	No	Yes	Yes
Solid residual	Ash	Char	Char

A separate dryer is not inherently required for an FBF incineration system. Drying occurs within the furnace if the combustion air is sufficiently preheated. The separate dryer adds complexity and additional reactor heat losses to pyrolysis and gasification systems. The target dryness depends on system supplier requirements, and ultimately, all water entrained with the sludge or biosolids is evaporated.

The thermal reactor treats the dried product downstream of a dryer in a pyrolysis or gasification system. In incineration and gasification, the air is introduced following the dryer, where present, for full or partial combustion, respectively. After the thermal reactor, a thermal oxidizer provides direct combustion of the fuel‐rich off‐gas from pyrolysis and gasification with a stream of air. This thermal oxidizer, or afterburner, is integral to an incinerating FBF and requires ample open space directly above the primary combustion zone (see Winchell et al., 2021 for a description of an FBF) and is, therefore, another simplifying advantage of an incineration system. However, combustion within a thermal oxidizer—required for pyrosis and gasification—improves process efficiencies through greater mixing between fuel and oxygen, limiting the amount of excess air, which acts as a heat sink.

At the outlet of the thermal oxidizer, flue gas is at its highest temperature and is the most practical point at which to recover heat or energy. The main application for recovered heat is maintaining the temperature within the thermal equipment to drive the process. In the case of an FBF, the recovered energy typically pre‐heats combustion air, achieving temperatures up to 675°C depending on the fuel characteristics of the sludge or biosolids (WEF, 2009). In pyrolysis or gasification reactors, the recovered heat from the thermal oxidizer helps sustain the temperature in the thermal reactor and evaporates water in the upstream dryer.

Heat energy exceeding process demands can be recovered but adds complexity to the system. Incinerators have been coupled with steam boilers, organic Rankine cycle (ORC) systems, and thermal oil heat exchangers to produce power or provide energy for other processes (Hoener et al., 2017). Pyrolysis and gasification systems can likewise be integrated with these systems, assuming adequate energy remains after process needs are met. An advantage for pyrolysis and gasification systems is the off‐gas produced, composed of non‐condensable mixtures of CO, CO_2_, H_2_, CH_4_, H_2_O vapor, and other simple hydrocarbons, which can be processed and used in a wide variety of secondary applications (Basu, 2013). The use of off‐gas also generates added complexity for processing and transporting the gas fuel to a final application. The extent and complexity of “external” energy recovery options were not evaluated to streamline this evaluation, and the discussion herein is limited to internal process energy demands.

All thermal processes viable for use at WRRFs require flue gas treatment before release to the atmosphere. System suppliers have a wide array of air pollution control (APC) equipment options for process and site‐specific emission criteria. The type of equipment and specific purposes are adequately described elsewhere for combustion systems (Niessen, 2002; WEF, 2009). Similar APC would be considered for pyrolysis and gasification systems though the sizing would differ based on gas flow rate. Vapors from a dryer require separate treatment or join the gas flow from the thermal reactor off‐gas before thermal oxidation. Ultimately treated flue gas flows through a stack at a required height before discharging to the atmosphere.

The thermal treatment produces a residual solid, that is, char from pyrolysis and gasification or ash from combustion. In an FBF incineration system, all ash flows out of the reactor with the flue gas (Winchell et al., 2021), which can lead to issues with the erosion of downstream equipment. Thus, pyrolysis and gasification systems may offer an advantage because char is often removed mechanically, resulting in a lower particulate loading in the flue gas, which reduces erosion and related issues.

### Operating conditions

A survey of potential suppliers was conducted for this study to document differences with currently available systems. Equipment suppliers were asked to provide operating conditions specific to their system for a case study example. Dewatered solids, generically representative of sludge or biosolids characteristics (Table 2), were presented to each supplier to assess each system on an equal basis.

**TABLE 2:** Baseline dewatered solids characteristics

Parameter	Units	Value
Total solids	Percent	28
Combustible solids (CS)	Percent	75
Higher heating value	kJ/dry kg CS	23,260
Ultimate analysis	Percent of combustible	
Carbon		57
Hydrogen		7
Oxygen		30
Nitrogen		5
Sulfur		1

Table 3 summarizes the information provided by the equipment suppliers. The unit feed rate ranged broadly for all technologies, with one of the pyrolysis vendors, Supplier 2, focusing on lower capacity systems. Recycled energy was characterized for the dryer and thermal reactor (i.e., incinerator, pyrolyzer, or gasifier). For comparison, recycled energy was solicited and normalized to the maximum capacity for each system. Because the incinerator dries feedstock in the reactor or furnace, no recycled energy is noted compared with pyrolysis and gasification technologies. The incinerator recycled energy comes from preheating combustion air with the hot flue gas to a temperature that eliminates the need for supplemental heat, otherwise known as autogenous operations. Thus, the incinerator requires less energy (recovered) to operate without supplemental heat and potentially provides a more significant opportunity for exporting energy to other processes, such as power production. The incinerator normalized operating motor load fell between the range of pyrolysis and gasification suppliers.

**TABLE 3:** Thermal treatment operating conditions

Parameter	Units	Supplier 1	Supplier 2	Supplier 3	Supplier 4	Supplier 5	Supplier 6
Process	—	Incineration	Pyrolysis	Pyrolysis	Gasification	Pyrolysis	Gasification
Unit feed rate range	Dry tonne/day	6.8–107	2.1–6.8	22–110	22–90	6.7–56.7	6.1–24.4
Dryer							
Type	—	N/A	Rotary cylinder	Belt in tandem with rotary drum	Rotary drum	Rotary drum	Rotary drum
Target total solids	Percent	N/A	80	90	90	60	92
Temperature	°C	N/A	65	80–105	87	800	535 inlet 100 outlet
Solids residence time	Min	N/A	3330	220	20	20	15
Evaporative capacity	kg H_2_O/h	N/A	720	14,500	13,600	4960	2720
Thermal efficiency	kJ/kg of H_2_O	N/A	1939	N/P	2775	2685	3400
Supplementary fuel	kJ/h per dry tonne/day	N/A	0	0	0	45,100	0
Recycled energy input^b^ ^,^ ^e^	kJ/h per dry tonne/day	N/A	296,000	N/P	416,200	168,000	281,100
Reactor							
Type	—	FBF	Inclined screw	Passive falling tower	Fluidized bed	Rotary kiln	Moving chain grate
Temperature	°C	700–800 (sand bed) 800–900 (freeboard)	620	950	680	650–850	750
Gas residence time	s	6–10	7–8.5	10	8–10	1.2	1
Solids residence time	min	<1	15	15	20	20	90
Stoichiometric air	—	1.4	0.0	0.0	0.32	0.0	0.3
Supplementary fuel	kJ/h	0	0	0	0	0	0
Recycled energy input^b^ ^,^ ^e^	kJ/h per dry tonne/day	190,300	76,300	N/P	0	190,400	0
Thermal oxidizer							
Type	—	Integral	Flameless direct fired	Regenerative thermal oxidizer	Direct fired	Staged air cyclone	Proprietary
Temperature	°C	N/A	980	850	980	850	1200
Gas residence time	s	N/A	2.5–3.5	2.5	1–2	2	2
Flue gas flow rate^b^	Nm^3^/h per dry tonne/day	470	180	N/P	400	330	300
Supplementary fuel^b^ ^,^ ^c^	kJ/h per dry tonne/day	N/A	0	0	0	2400	0
Stoichiometric air	—	N/A	1.15	N/P	2.9	N/P	1.15
Energy recovered^a^	Percent of available from flue gas sensible heat	30	71	70^f^	75	65^g^	79
Major motor requirements^b^	kW/dry tonne/day	8.4	23.5	1.8	15.7	4.0	4.7
Solid residual							
Production^d^	Percent of dry feed	25	45	N/P	27	35.8	25
Combustible fraction	Percent	0	10	N/P	14	16–30	0
Carbon content	Percent	0	30	<2	14	15–25	0
Nitrogen content	Percent	0	3	Minimal	Minimal	0	0

Abbreviations: N/A, not applicable; N/P, not provided.

^a^ Amount required to self‐sustain process.

^b^ Normalized to feed rate at maximum size offered by the equipment supplier.

^c^ Natural gas equivalent.

^d^ Based on solid characteristics presented in Table 2.

^e^ Heat recycled to sustain the process.

^f^ Author‐calculated value based on 5‐MW power generation quoted by the supplier at 204 dry tonne/day at 20% total solids and assuming 40% power production efficiency, combustible solids, and heating values per Table 2. Value is conservative as it ignores energy radiation losses, latent heat of vaporization for water resulting from the combustion of off‐gas, and heat demand to raise combustion air to process temperature.

^g^ Supplier recycles a portion of energy as cleaned off‐gas in addition to heat recovery from the flue gas. Value estimated using the heating value of cleaned off‐gas, actual energy recovered percentage is higher if the latent heat of water vapor from combustion is included but was not available.

The solids retention time in the pyrolysis and gasification reactors is high compared with an incinerator. Therefore, pyrolysis and gasification technologies may require a larger footprint depending on the configuration to process the same feed rate.

Based on the amount of oxygen required over the stoichiometric combustion demand for efficient processing, stoichiometric air is expectedly high in the incinerator compared with reductive technologies. Incineration requires a significant amount of air, or oxygen, above the theoretical stoichiometric amount to combust the fuel efficiently. This excess overcomes mixing inefficiencies in the furnace, ensuring the fuel adequately contacts oxygen for complete combustion. FBF incinerators used at WRRFs include 40% excess air (WEF, 2009). By contrast, pyrolysis and gasification reactors intentionally operate at substoichiometric oxygen rates (Table 3). Currently offered systems process the off‐gas through the thermal oxidizer. As a result, the combustible gas is more efficiently mixed with oxygen than incineration of a solid and gas phase mixture. Furthermore, pyrolysis and gasification systems produce a char often with some combustible fraction yet present, directly reducing the flue gas flow compared with an incineration process that intentionally converts all combustible material. Normalized flue gas flow rates, after the thermal oxidizer, are lower for pyrolysis and gasification systems.

As expected, char production from pyrolysis or gasification as a percentage of dry feed is higher than incineration ash, and the resulting material includes higher carbon content except when intentionally converted to ash to minimize the solid residual, as was the case of Supplier 6. Phosphorus was not listed because the content in the solid residual depends on the feedstock concentration, which remains with the solids (i.e., char and ash).

### Major outputs

Regardless of the thermal technology or site‐specific considerations, each process results in solid and gas‐phase emissions. The solid ash or char is collected from the thermal reactor or APC equipment, whereas gas‐phase emissions are ultimately emitted to the atmosphere through the process stack. During pyrolysis and gasification, off‐gas production increases as temperatures increase, whereas char yields decrease due to the loss of volatile components (Chen et al., 2014; Yuan et al., 2015). Nearly all carbon is combusted, and volatile components are lost during incineration, resulting in ash comprised of inorganic minerals dominated by silicon (Si), aluminum (Al), iron (Fe), and phosphorus (P) oxides along with alkaline earth minerals (Li et al., 2018; Tempest, 2013).

#### Solid residual

Although a regulatory framework has existed under the USEPA 40 C.F.R. § 503 (1993) since the 1990s that allows for land application, incinerator ash has been mostly landfilled and not beneficially reused. Beneficial reuse of ash as a potential ingredient for construction materials, including cement and bricks, has been investigated, but varying particle size and morphology have limited its use (Donatello & Cheeseman, 2013; Naamane et al., 2016; Smol et al., 2015). Char is a beneficial soil amendment; however, properties important to soil quality, such as pH, cation exchange capacity, and nutrients, can vary widely and are dependent on feedstock characteristics and pyrolysis temperatures (Al‐Wabel et al., 2018). Additionally, although char can be described as a material derived from sludge or biosolids, as defined in USEPA's biosolids regulations, 40 C.F.R. § 503, it may be a lengthy process for producers to receive recognition of char as an Exceptional Quality or Class A biosolids product from regulators and use it as a soil amendment.

Increasing pyrolysis temperatures increase sludge and biosolids‐derived char pH, surface area, pore‐volume, total P (TP), and potassium (K) concentrations, and decrease N concentrations (Table 4). Therefore, targeting specific char characteristics could be achieved by using select temperatures. Nutrients, such as N, P, K, and S, in char and ash for land application reuse, follow different fate pathways during thermal processing. For example, up to 40% of the N in biosolids can be lost to the gas phase, primarily as ammonia and hydrogen cyanide, at temperatures up to 800°C (Chen et al., 2011; Wei et al., 2015). Similarly, Hossain et al. (2011) found that up to 40% of S volatilized with increasing temperatures. P and K, however, become concentrated in char and incinerator ash on a mass concentration basis as biomass is lost (Table 4; Egle et al., 2016; Lu et al., 2013; Yuan et al., 2015).

**TABLE 4:** Chemical properties of sludge and biosolids‐derived char and incinerator ash^a^

Parameter	Unit	Biosolids/sludge	Char			Incinerator ash		Land application limits EQ/ceiling
			500°C	600°C	700°C			
pH^b^ ^−^ ^j^		4.4–7.2	6.5–9.8	8.1–12	8.4–12			
Surface area^b^ ^,^ ^e^ ^−^ ^j^	m^2^/g	2.2–18	3.2–52	12–27	27			
Carbon^b^ ^−^ ^f^ ^,^ ^h^ ^,^ ^j^	wt. %	21–38	18–21	20–21	20			
Nitrogen^b^ ^−^ ^f^ ^,^ ^h^ ^−^ ^j^	wt.%	3.0–5.4	1.8–3.1	1.5–2.7	0.91–1.2			
Phosphorus^b^ ^,^ ^e^ ^,^ ^f^ ^,^ ^h^ ^−^ ^j^	wt. %	1.5–5.2	3.6–6.1	4.5	4.9	7.9 (1.5–13)	7.6 (1.0–14)	
Potassium^b^ ^,^ ^e^ ^,^ ^f^ ^,^ ^h^ ^−^ ^j^	wt. %	0.08–0.75	0.13–1.0	0.26–1.3	1.7	0.9 (<0.006–1.7)	1.0 (0.1–3.7)	
Sulfur^b^ ^−^ ^f^ ^,^ ^j^	wt. %	0.67–5.2	0.50–5.9	0.55–0.87	6.2	1.0 (0.3–6.9)	1.3 (0.1–1.5)	
Zinc^b^ ^−^ ^j^	mg/kg	306–2580	411–2822	1090‐3368	1090‐2175	2534 (552–5515)	2950 (600–9333)	2800/7500
Copper^b^ ^−^ ^j^	mg/kg	115–1218	138–1674	209–1697	227–1500	785 (162–3467)	1262 (470–6991)	1500/4300
Lead^b^ ^−^ ^j^	mg/kg	20–3740	93–5120	111–5250	132–5200	117 (<3.5–1112)	298 (38–3000)	300/840
Nickel^c^ ^−^ ^e^ ^,^ ^g^ ^−^ ^j^	mg/kg	23–112	35‐292	101–219	103–195	75 (8.2–501)	122 (40–625)	420/420
Cadmium^c^ ^,^ ^e^ ^−^ ^j^	mg/kg	BDL–169	3.2–235	229	3.2–123	2.7 (<0.1–80)	7.7 (0.5–128)	39/85
Arsenic^c^ ^,^ ^e^ ^,^ ^h^ ^,^ ^i^	mg/kg	<3–26	<3–32	35	<3–37	14 (4.2–124)	15 (1.6–40)	41/75
Chromium^b^ ^−^ ^e^ ^,^ ^h^ ^−^ ^j^	mg/kg	20–449	61–1065	106–1374	83–103	160 (58–1502)	411 (15–5019)	
Reference(s)		^b^ ^−^ ^i^	^b^ ^−^ ^d^ ^,^ ^f^ ^−^ ^j^	^d^ ^,^ ^f^ ^,^ ^i^	^c^ ^,^ ^i^	Krüger et al. (2014)^k^	Ma and Rosen (2021)^l^	USEPA (1993)

Abbreviations: BDL, below detention limit; EQ, exceptional quality.

^a^ Showing range of reported values for biosolids/sludge and different pyrolysis temperatures.

^b^ de Figueiredo et al. (2019).

^c^ Hossain et al. (2011).

^d^ Jin et al. (2016).

^e^ Khan et al. (2013).

^f^ Lu et al. (2013).

^g^ Méndez et al. (2012).

^h^ Song et al. (2014).

^i^ Yuan et al. (2015).

^j^ Chagas et al. (2021).

^k^ Incinerator ash elemental values reported as median with the minimum and maximum in parentheses from sample population of 252.

^l^ Incinerator ash elemental values reported as median with the minimum and maximum in parentheses from sample population between 24 and 101 depending on the element.

TP concentrations in biosolids‐derived char increased by 40% to 50% at 700°C, indicating P was associated with the inorganic fraction of biosolids (Hossain et al., 2011; Yuan et al., 2015). Due to less biomass lost during pyrolysis, TP concentrations in char are lower than concentrations in incinerator ash, which can be as high as 14% (Table 4; Krüger et al., 2014; Ma & Rosen, 2021). High concentrations of TP in incinerator ash may increase its potential as a fertilizer, but it is bound in mineral forms that are not readily available to plants (Gorazda et al., 2017; Ma & Rosen, 2021). High P concentrations are also a challenge for reuse as a construction material due to detrimental effects on the strength of cement (Ottosen et al., 2013; Ottosen et al., 2020). In contrast, P in sludge and biosolids‐derived char, though still in inorganic form, is more soluble and plant available (Yang et al., 2021).

The pyrolysis, gasification, and incineration of sludge and biosolids volatilize a small portion of heavy metals in the feedstock; however, the remainder is concentrated in the residual ash and char due to loss of biomass (Chanaka Udayanga et al., 2018). Similar to TP concentrations, pyrolysis of sludge and biosolids results in lower concentrations of metals than in incinerator ash (Table 4) and a reduction of leaching and bioavailability to plants relative to the feedstock (Jin et al., 2016; Lu et al., 2016). Méndez et al. (2012) demonstrated that sludge pyrolysis decreased the plant available and mobile forms of nickel (Ni), zinc (Zn), copper (Cu), and lead (Pb). When blended with agricultural soil, the leaching of Cu, Ni, cadmium (Cd), and Zn from char was lower relative to raw sludge. This enhanced sorption is attributed to the large surface area, porous structure, and complexation with surface functional groups and has also been shown to reduce the uptake of PAHs by plants and remove micropollutants, including metals, hormones, and pharmaceuticals and personal care products (PPCPs) from wastewater (Khan et al., 2013; Kimbell et al., 2018; Tan et al., 2015; Tong et al., 2019). Although incinerator ash has agronomic value as a soil amendment due to high P concentrations and alkalinity, land application is expected to be further limited due to permitting hurdles. Methods to remove P so that ash can be processed into bricks and other construction materials (Ottosen et al., 2020) are under development.

#### Air emissions

Contemporary APC systems can be configured to meet current regulatory emissions limits for any thermal technology discussed, but pyrolysis and gasification systems offer distinct advantages. The two primary advantages are the volume and mass of flue gas treated and the thermal chemistry.

Table 3 and the associated discussion indicate that pyrolysis and gasification systems result in a significantly reduced flue gas flow rate compared with incineration. Therefore, APC systems designed for comparatively smaller flue gas flows from pyrolysis and gasification systems are smaller and cost less.

Reduced nitrogen oxide (NO_X_) emissions from pyrolysis and gasification processes provide a significant advantage over incineration. NO_X_ consists of both nitric oxide (NO) and nitrogen dioxide (NO_2_) and requires the presence of oxygen to form in a high‐temperature process (Niessen, 2002). Thermal and fuel‐bound mechanisms produce NO_X_ in thermal processes. The thermal mechanism reacts nitrogen gas (N_2_) and oxygen at high temperatures to produce NO. When processing WRRF sludge or biosolids, the thermal mechanism only becomes significant at a temperature over 1093°C (WEF, 2009). The fuel‐bound mechanism uses the nitrogen in the fuel, typically 3%–6% of WRRF solids (WEF, 2009), to react with oxygen to produce NO_X_. Both mechanisms require free oxygen to proceed and, in pyrolysis and gasification systems that limit oxygen, NO_X_ formation is suppressed. Instead, fuel‐bound nitrogen primarily converts to N_2_ and ammonia (Basu, 2013).

In the thermal oxidation step of the pyrolysis and gasification systems, the amount of excess oxygen introduced can be significantly less than for incineration (Table 3). Therefore, NO_X_ emissions are lower compared with incineration. Pyrolysis and gasification systems may also sequester some nitrogen in the char (Tables 3 and 4), further limiting NO_X_ emissions. Ultimately, the NO_X_ emissions limits set by regulatory agencies dictate whether thermal processes require APC, typically including selective noncatalytic reduction (SNCR) using ammonia or urea. None of the pyrolysis or gasification system suppliers indicated the need for an SNCR system. For FBF SSIs, the limit specified under the SSI air emissions regulations 40 C.F.R. § 60 (2011) of 30 parts per million by dry volume (ppm_vd_) at 7% oxygen can be met at some facilities without controls. Others use SNCR, as experienced by the authors.

The thermal processes reviewed herein offer no comparative advantage for other common air pollutants. S and chlorine (Cl) associated with the sludge or biosolids essentially become regulated pollutants such as sulfur oxides (SO_X_) and hydrochloric acid (HCl), respectively. In incineration systems, S in the feedstock is oxidized to SO_X_ (Niessen, 2002). In contrast, in pyrolysis and gasification systems, the flue gas generally contains hydrogen sulfide (H_2_S) or carbonyl sulfide (COS) (Basu, 2013). Given the close coupling of the thermal oxidizer with pyrolysis and gasification systems, these reduced S compounds are oxidized to SO_X_ and thus emit the same load as incinerators. However, some sulfur may remain with the char during pyrolysis (Table 4). In contrast, others have noted that up to 96% of the sulfur in gasification feedstock evolves into the off‐gas (Higman & van der Burgt, 2008). The Cl present in the sludge or biosolids will likewise primarily result in HCl emissions from incineration (Niessen, 2002; WEF, 2009), gasification (NETL, 2021), and pyrolysis (Karama et al., 2013).

The fate of metals through thermal technologies depends on whether they remain with the solid (char or ash) or volatilize. Because thermal processes neither create nor destroy metals, they are equally subjected to handling the pollutant load in the sludge or biosolids and meeting the regulatory requirements. The volatility of various metals may offer some benefit to operating a thermal reactor at lower temperatures (Gerstle & Albrinck, 1982; Niessen, 2002; WEF, 2009). Arsenic (As), Cd, mercury (Hg), selenium (Se), and Zn represent typical metals of interest that readily volatilize in thermal processes operating at reactor temperatures noted in Table 3. Beryllium (Be), chromium (Cr), Cu, and Ni remain in particulate form. Therefore, control of their air emissions is dictated by capturing particulate matter (PM). The volatility temperature of lead (Pb) (627°C in elemental form) may exceed the thermal reactor operating temperature in pyrolysis and gasification systems (Niessen, 2002). Thus, Pb may remain with the char or ash, and additional research is needed to characterize the content and potential leachability in char reuse applications building off previous efforts (Jin et al., 2016; Lu et al., 2016; Méndez et al., 2012).

Particulates emitted with the flue gas require removal to meet air emissions regulations. In FBFs, all the ash flows out of the furnace with the flue gas requiring a robust APC system that removes PM (Winchell, Ross, et al., 2022). Conversely, pyrolysis and gasification systems generally remove the char from the thermal reactor mechanically, reducing the potential particulate load in the flue gas. In the authors' experience, particulate control device suppliers design their equipment for a specific percent removal. Thus, the APC system design may offer cost savings or reduce PM emissions with a lower particulate load. The tradeoff then becomes the costs and complexity of the char removal system.

All three technologies evaluated use a thermal oxidizer, internal or external to the thermal reactor, to oxidize products of incomplete combustion (PIC). Whether PICs are measured as total hydrocarbons (THC), volatile organic compounds (VOC), or CO, the energy inherent to sludge or biosolids can support temperatures in the thermal oxidizers to achieve similar destruction performance (WEF, 2009). Emissions of polychlorinated dibenzo‐p‐dioxins (PCDD) and polychlorinated dibenzofurans (PCDF)—a subset of VOCs that received much interest over the last few decades—from the three technologies can likewise be expected to generate similar performance based on the presence of a thermal oxidizer. Incineration would likely outperform pyrolysis and gasification systems without the external thermal oxidizer. Conesa et al. (2020) observed reduced PCDD/PCDF emissions from thermal processes treating sludge as the oxygen stoichiometric ratio increased, with the lowest emissions occurring at the highest oxygen ratio analyzed (120%). The overall emissions of PCDD/PCDF will, more importantly, be dictated by the thermal oxidizer performance and downstream conditions. McKay (2002) extensively reviewed PCDD/PCDF occurrence in municipal solid waste incineration emissions, noting that the formation of these compounds occurs at approximately 400°C. If these conditions persist downstream of the thermal oxidizer, PCDD/PCDF may reform and should be avoided where practical unless specific APC equipment is included to capture these compounds. Given that all the thermal technologies recover heat from the flue gas to support the process and potentially transfer heat for external use, the emission of PCDD/PCDFs cannot be distinguished among the thermal technologies.

As noted, the emissions from these thermal processes are controlled to meet the regulatory requirements. A subsequent section covers permitting in more detail. Table 5 was developed using the emissions limits from SSI air emissions regulations 40 C.F.R. § 60 (2011), specifically the new FBF category. Permitted emissions for three pyrolysis and gasification facilities were converted where possible to the concentration‐based limits for SSIs using various assumptions footnoted in Table 5 for comparison. The current emission limits are more lenient because the pyrolysis and gasification technologies have not been classified under SSI air emissions regulations 40 C.F.R. § 60 (2011). However, recent action by the USEPA may promulgate new future regulations specific to pyrolysis and gasification systems, including those processing sludge or biosolids. At this point, the USEPA is collecting information and comments related to the applicability of 40 C.F.R. § 60 to these technologies to classify them more clearly (USEPA, 2021b).

**TABLE 5:** Allowable stack emissions for various thermal processes

Type^a^	FBF (SSI)	Pyrolysis^b^	Gasification^c^	Gasification^d^
PM mg/dry standard cubic meter (dscm)	9.6	13^e^ ^,^ ^f^	381	—
CO ppm_vd_	27	235^g^	79	—
NO_X_ ppm_vd_	30	143^g^	545	51
SO_2_ ppm_vd_	5.3	26^g^	205	71
Cadmium mg/dscm	0.0011	0.0014^e^	—	—
HCl ppm_vd_	0.24	17^e^	—	—
Mercury mg/dscm	0.0010	0.016^e^	12	—
Lead mg/dscm	0.00062	0.021^e^	—	—
PCDD/PCDF (total mass) ng/dscm	0.013	0.0033^e^ ^,^ ^h^	—	—

^a^ Pollutant concentration normalized to 7% O_2_.

^b^ Bay Area Air Quality Management District (BAAQD, 2021).

^c^ Permit values are presented as mass loads emitted (State of Tennessee, 2017b). Converted to concentration basis using stack test (State of Tennessee, 2017a) flue gas flow pro‐rated to permit basis using the measured (18.1 million kJ/h) and permitted (30.6 million kJ/h) heat inputs. The resulting values normalized to 7% O_2_ from stack test measured O_2_ (15.19%).

^d^ Based on the permit to construct the facility, only values for NO_X_ and SO_2_ were compatible with SSI regulations, whereas other pollutant limits could not be converted with information available (State of New Jersey, 2020).

^e^ Converted from permit mass load, yearly chronic values for all but PM, which was hourly based, using flue gas flow rate calculated from mass load and concentration limits for CO, NO_X_, and SO_2,_ assuming resulting value at 7% O_2_.

^f^ Based on the permitted PM10 value.

^g^ Converted from the permitted basis of 3% O_2_.

^h^ Permit value includes dioxin‐like polychlorinated biphenyls as the 2,3,7,8‐PCDD equivalent.

### Unregulated chemical removal and destruction

With increasing public concerns surrounding PFAS and other emerging pollutants, WRRF owners and operators need technologies to destroy these chemicals, which may occur in pyrolysis and gasification systems. Unregulated chemicals, including PFAS, PPCPs, steroids, hormones, and other emerging pollutants, have been detected in wastewater effluent, sludge, and biosolids (McClellan & Halden, 2010; Patel et al., 2019; USEPA, 2009). Whereas conventional wastewater treatment processes may transform some of these chemicals, others pass through or partition into sludge and biosolids (Kinney et al., 2006; Luo et al., 2014; Spongberg & Witter, 2008; Walters et al., 2010). The significant reduction or complete removal of these chemicals from sludge or biosolids‐derived char is necessary for WRRFs to enable land application and beneficial reuse.

The fate of emerging pollutants has been discussed for pyrolysis systems (Winchell, Ross, et al., 2022) in detail. For example, Mercl et al. (2021) tested 69 pharmaceuticals from 27 drug classes in biosolids finding undetectable levels in the char after pyrolysis at 420°C. Williams et al. (2021) found three of 28 targeted PFAS species could be detected in char after pyrolysis at 500°C and none at 700°C. The same study analyzed the pyrolysis off‐gas stream for 31 specific PFAS and found a combined mass removal efficiency of 84.4 and 95.6% of measured PFAS when including that found in the char at the two experimental temperatures, respectively. Thoma et al. (2021) evaluated PFAS through a full‐scale pyrolysis system processing dried biosolids, observing 81.3 to 99.9% removal for 21 compounds. Direct combustion of pyrolysis and gasification off‐gases provides a promising opportunity to oxidize fully any PFAS transformation products remaining. Recent emission results at the Saint‐Gobain Performance Plastics (SGPP) and Chemours industrial facilities (Beahm, 2019; Focus Environmental Inc., 2020) established 99.99% destruction efficiency of specific PFAS at the latter facility when operating at 980°C and residence times of 0.75 to 1.2 s.

There is a paucity of literature on the fate of most emerging pollutants through SSIs, although PFAS research efforts are increasing. Winchell et al. (2021) compiled the current body of published information and concluded that SSIs operate under conditions expected to destroy PFAS, but further research is ongoing to establish the destruction and removal efficiency.

Although some work has been advanced to fully elucidate the fate and transformation products of emerging pollutants in the residuals, oils, and gases from thermal treatment technologies to enable proper management, additional research is needed. Future research must address the fate through the treatment systems and the analytical science to detect and measure broad classes of chemicals at toxicologically relevant levels.

### Permitting

Given the limited history of sludge or biosolids processing, permitting a gasification or pyrolysis facility differs significantly from an SSI. The Clean Water Act and Clean Air Act have sections specific to “incineration” of “sewage sludge.” Further, both Acts include definitions specifying combustion of the sludge or biosolids as a critical characteristic of incineration, 40 C.F.R. § 60 (USEPA, 2011). However, there are no specific references to pyrolysis, gasification, or associated thermal oxidizers, and permitting requirements are subject to a case‐by‐case determination (Hambrick, 2021). To date, pyrolysis and gasification facilities have avoided the “incineration” designation (Aries Clean Technologies, 2021; USEPA, 2013, 2016) and the associated regulatory requirements. In particular, the SSI air emissions regulations 40 C.F.R. § 60 (2011) require SSIs to obtain a Title V operating permit without exception; they impose concentration emission limits for nine pollutants, requirements for conducting and documenting certified staff training, and process documentation. Thus, the current regulatory framework provides pyrolysis and gasification systems a distinct advantage over SSIs if they can continue avoiding the “incineration” designation.

Despite the tentative exemption from SSI regulations, pyrolysis and gasification systems must undergo an air permitting process. The process includes identifying the magnitude of potential emissions, facility classification, and attainment status of the installation location in support of a construction permit application and subsequent operating permit. WEF (2009) provides a general overview of the permitting process for SSIs, which would generally apply to pyrolysis and gasification systems, in addition to the USEPA site‐specific applicability determination ruling.

### Cost comparisons

**FIGURE 4:**
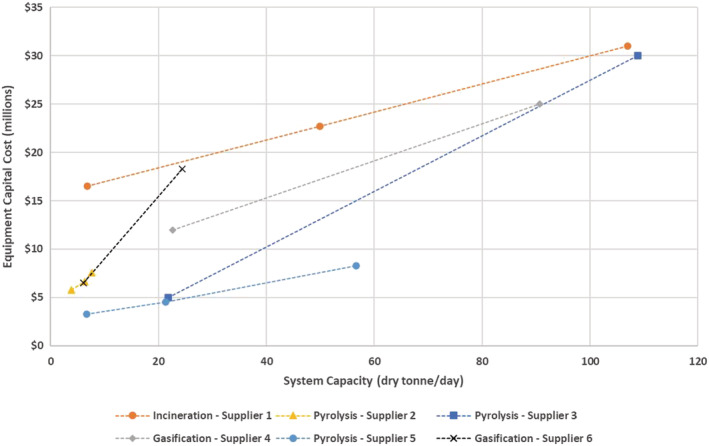
Equipment capital costs for incineration, pyrolysis, and gasification systems for various system capacities

Suppliers provided planning level capital costs for a single train of equipment from the point of dewatered sludge or biosolids delivery to the stack, including a handling system for residual solids. Figure 4 illustrates costs provided from all responding technology suppliers, spanning the capacity ranges of individual suppliers. Incineration Supplier 1's system included piston pumps and hoppers for conveying dewatered sludge or biosolids. The cost for this equipment was not removed due to the generality of the quote, but a previous proposal from the same supplier identified roughly $3 million at the 110 dry tonne/day level for this equipment. One pyrolysis supplier (Supplier 3) also included internal combustion engines for producing power from the pyrolyzer off‐gas, given this is part of their core offering. The engine costs were also not removed, but the price can be expected to be several million dollars at the higher end of the capacity range. Costs were limited to equipment because installation costs are unpredictable until a specific site is selected. The pyrolysis and gasification technologies offer a cost–benefit at lower processing capacities but become comparable with incineration at the 110 dry tonnes/day capacity. The reason for the reduced costs at lower capacity for pyrolysis and gasification is not apparent, but the dryer or APC equipment used by the pyrolysis and gasification suppliers may be common at this scale and thus more affordable. Given the cost advantages of pyrolysis and gasification technologies in small to mid‐size treatment facilities, this is likely where these technologies will enter the wastewater market.

Project costs will, of course, hinge on other site‐specific factors, in addition to equipment costs. One of the more significant costs is the structure used to house equipment and land requirements for the facility. This evaluation did not attempt to characterize the footprint for the various technologies. For suppliers offering similar scale capacities, the footprint requirements are arguably similar. For example, where incineration does not require a dryer, that footprint can be occupied by large APC equipment. However, smaller‐scale suppliers are at a competitive disadvantage for higher total capacity facilities as multiple trains with individual space requirements may quickly expand beyond the footprint of higher capacity offerings.

## KNOWLEDGE GAPS AND FUTURE RESEARCH

The level of interest in pyrolysis and gasification within the wastewater community appears to be increasing based on the authors' experience. A formal survey of a diverse sample of WRRF utilities documenting this interest may invigorate research efforts and justify the funding required. The following discussion identifies key areas of research for validating these technologies as alternatives to the historically proven incineration process.

Two critical knowledge gaps exist regarding pyrolysis or gasification applications at WRRFs, perhaps the long‐term operation being the foremost. Contemporary installations include three active or recently decommissioned facilities with at most 5 years of operation (Winchell, Ross, et al., 2022). The long‐term durability of components subjected to high temperatures and the potentially abrasive nature of the materials being processed can lead to significant maintenance requirements anecdotally supported by the authors' experience with incineration systems. Winchell, Ross, et al. (2022) also discussed some of the challenges experienced by past versions of the pyrolysis and gasification technologies including material handling difficulties. Contemporary systems need to prove the equipment employed reliably handles sludge or biosolids and the resulting residuals. Finally, the energy balance based on extensive operating data must validate the values presented in Table 3. Although the operating history may not exist in the United States, the currently active installations can be monitored for at least 10 years of operation from a mechanical durability viewpoint but more immediately when considering material handling and energy balance.

The second critical knowledge gap to note is the lack of emerging contaminant fate through these processes. Several studies are currently underway evaluating the fate of PFAS through SSIs (MacGregor, 2021; WRF, 2022). The WRRF community requires similar research on pyrolysis and gasification processes to build on initial efforts discussed previously (Thoma et al., 2021; Williams et al., 2021). Because of their unmatched thermal stability (Winchell et al., 2021), the fate of PFAS through these processes will provide indirect evidence for other organic compounds less resistant to thermal decomposition. Nevertheless, a thorough evaluation of various PPCPs, steroids, and hormones in contemporary high‐temperature processes is required for assuring the quality of the residual products and confirming environmental impacts.

A parallel and similar track of research shall include the drying process critical to the success of pyrolysis and gasification systems. Winchell, Ross, et al. (2022) discussed the importance of drying for these high‐temperature systems and the lack of a reliable technology to dry sludge compared with digested biosolids. Overcoming this hurdle would broaden the applicability of these pyrolysis and gasification technologies. Furthermore, advancements in drying technology (WEF, 2014) may reduce the energy demand and improve the overall energy balance but the capabilities of current commercial offerings has not been comprehensively documented.

## CONCLUSIONS

Several suppliers have recently emerged in the wastewater market to offer pyrolysis and gasification technologies to process sludge and biosolids from WRRFs. Undeniably, these technologies reduce the final mass that a facility operator would need to manage. Further, the final residuals are valuable for beneficial reuse, potentially leading to a zero‐waste process, although the fate of specific chemical contaminants requires further study. Finally, with current knowledge of operating conditions, some transformation and destruction of emerging pollutants occur, potentially yielding a relatively clean solid residual with little to no air emissions. These characteristics have led to strong interest in pyrolysis and gasification as alternatives to incineration in the wastewater industry.

In summary, primary advantages of pyrolysis and gasification systems versus a contemporary incinerator identified by this investigation include reduced combustion air requirements, reduced flue gas flows resulting in smaller emissions control equipment, potentially carbon‐rich solid residuals, lower unabated NO_X_ emissions, potentially lower PM emissions depending on reactor configuration, and lower equipment costs at the reduced processing capacities. These advantages need to be weighed against the added complexity of a dryer, thermal oxidizer, and energy recovery. Finally, and possibly most importantly, pyrolysis and gasification systems are exempted from SSI regulations and emissions limits resulting in more streamlined permitting and operations, although a current USEPA regulatory review may result in changes. Altogether, pyrolysis and gasification show promise as alternatives to incineration for some, particularly small to mid‐sized facilities, and the current and contracted‐to‐build installations will, over time, provide valuable information on real‐world operating and maintenance requirements. We may be on the cusp of a technological evolution if the historical issues of pyrolysis and gasification are overcome.

## AUTHOR CONTRIBUTIONS

**Lloyd Winchell:** Conceptualization; data curation; formal analysis; investigation; methodology; project administration; supervision; visualization. **John Ross:** Conceptualization; data curation; formal analysis; investigation. **Dominic Brose:** Conceptualization; data curation; formal analysis; investigation; visualization. **Thaís Pluth:** Conceptualization; data curation; formal analysis; investigation; visualization. **Katherine Bell:** Conceptualization; project administration.

## Data Availability

The data that support the findings of this study are available from the corresponding author upon reasonable request.
